# Macrophage Inhibitory Cytokine 1 Biomarker Serum Immunoassay in Combination with PSA Is a More Specific Diagnostic Tool for Detection of Prostate Cancer

**DOI:** 10.1371/journal.pone.0122249

**Published:** 2015-04-08

**Authors:** Ji Li, Robert W. Veltri, Zhen Yuan, Christhunesa S. Christudass, Wlodek Mandecki

**Affiliations:** 1 PharmaSeq, Inc., Monmouth Junction, New Jersey, United States of America; 2 Johns Hopkins University School of Medicine (JHUSOM), Baltimore, Maryland, United States of America; Deutsches Krebsforschungszentrum, GERMANY

## Abstract

**Background:**

Prostate cancer (PCa) is the most common malignancy among men in the United States. Though highly sensitive, the often-used prostate-specific antigen (PSA) test has low specificity which leads to overdiagnosis and overtreatment of PCa. This paper presents results of a retrospective study that indicates that testing for macrophage inhibitory cytokine 1 (MIC-1) concentration along with the PSA assay could provide much improved specificity to the assay.

**Methods:**

The MIC-1 serum level was determined by a novel p-Chip-based immunoassay run on 70 retrospective samples. The assay was configured on p-Chips, small integrated circuits (IC) capable of storing in their electronic memories a serial number to identify the molecular probe immobilized on its surface. The distribution of MIC-1 and pre-determined PSA concentrations were displayed in a 2D plot and the predictive power of the dual MIC-1/PSA assay was analyzed.

**Results:**

MIC-1 concentration in serum was elevated in PCa patients (1.44 ng/ml) compared to normal and biopsy-negative individuals (0.93 ng/ml and 0.88 ng/ml, respectively). In addition, the MIC-1 level was correlated with the progression of PCa. The area under the receiver operator curve (AUC-ROC) was 0.81 providing an assay sensitivity of 83.3% and specificity of 60.7% by using a cutoff of 0.494 for the logistic regression value of MIC-1 and PSA. Another approach, by defining high-frequency PCa zones in a two-dimensional plot, resulted in assay sensitivity of 78.6% and specificity of 89.3%.

**Conclusions:**

The analysis based on correlation of MIC-1 and PSA concentrations in serum with the patient PCa status improved the specificity of PCa diagnosis without compromising the high sensitivity of the PSA test alone and has potential for PCa prognosis for patient therapy strategies.

## Introduction

Prostate cancer (PCa) is the most common malignancy among men in the United States, with 238,590 newly diagnosed cases and 29,720 deaths in 2013 [1]. Prostate-specific antigen (PSA) screening in the USA [2] has revolutionized the management of PCa over the past two decades, especially with regards to early detection, greatly improving the chances of a curative treatment [3]. However, a new problem emerged over the years: overdiagnosis and overtreatment of PCa [4, 5]. Overdiagnosis is estimated to constitute about 23–56% of cases, resulting in significant overtreatment. Approximately 60–80% of elevated serum PSA findings are false-positives, as determined by prostate biopsy, thus demonstrating the inability of PSA alone to discriminate between clinically significant PCa and benign diseases [3, 6]. Various computational derivative PSA methods, like PSA density (PSA level divided by prostate volume), PSA transition zone density, PSA velocity (change of PSA over time) and age- or race-specific reference ranges, have been developed to address the rate of false-negatives and false-positives, but these approaches do not always live up to expectations [7–11]. As a matter of fact, no single serum biomarker including PSA and its derivatives can currently fulfill the clinical needs of both high sensitivity and specificity. In this study, we developed an innovative p-Chip-based immunoassay that combines PSA levels and those for macrophage inhibitory cytokine 1 (MIC-1).

MIC-1, or growth differentiation factor 15 (GDF-15) or non-steroidal anti-inflammatory drugs (NSAIDs) activated gene (NAG-1), is a protein belonging to the transforming growth factor beta superfamily that has a role in regulating inflammatory and apoptotic pathways in injured tissues and during disease processes. MIC-1 is overexpressed in many patients with common cancers including those of the prostate and can be further induced by cancer therapies including surgery, chemo- and radiotherapy of prostate, colon and breast cancer [12, 13]. MIC-1 is linked to cancer in general and tumor expression of MIC-1 is often reflected in its blood levels, which increase with cancer development and progression [14, 15], generally in proportion to the stage and extent of disease. Previous work has suggested that in established PCa, MIC-1mRNA expression is higher in Gleason score ≥7 tumors compared with lower-grade lesions [16]. MIC-1 is highly expressed in the human PCa cell line LNCaP [17], is found in high-grade prostatic intraepithelial neoplasia and in cancer cells, but not in normal cells [18].

The p-Chip technology used in this study has been used in cell-based [19], nucleic acid [20] and protein [21] assays. The p-Chip is a passive, ultra small, integrated circuit that can transmit its unique identification code (ID) via radio frequency (RF) when stimulated by modulated laser light. The p-Chip can be derivatized with an appropriate biomarker probe, such as oligonucleotides or antibodies, to construct highly specific assays. Results are automatically determined on a custom fluidic analyzer (flow reader), similar to a flow cytometer which decodes the ID of each p-Chip and correlates it with the fluorescence intensity indicative of the concentration of the biomarker on each chip. The flexibility of the p-Chip-based platform easily allows for the adaptation of assays to any number of capture antibody probes, and to add or subtract probes as the relevance of markers evolves with additional discoveries.

**Fig 1:**
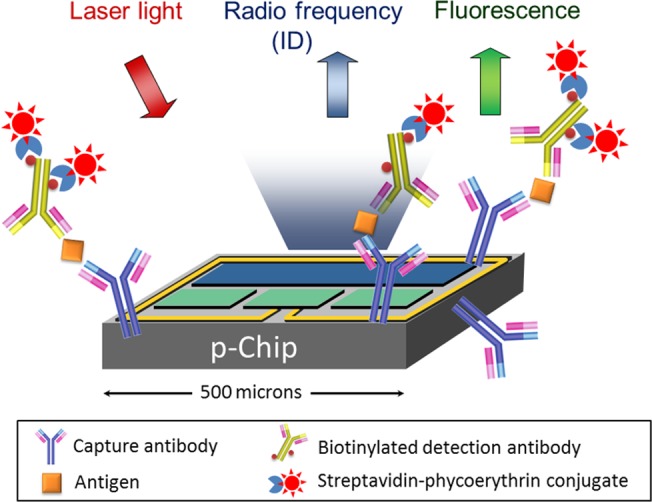
p-Chip-based immunoassays.

In this study, we developed a p-Chip-based MIC-1 immunoassay (Fig 1) and a method to diagnose prostate cancer involving a comparison of the PSA and MIC-1 levels with a reference. We found that the novel method greatly improved the clinical specificity of PCa determination without compromising the high sensitivity of the currently used PSA assay.

## Materials and Methods

The research was approved by the Johns Hopkins University School of Medicine (JHUSOM) IRB: The RW Veltri (P.I.) eIRB2 NA_00020740 entitled “Multi-transponder-based Prostate Cancer Multiplex Assay”. The project was classified as "Exempt".

### Antibodies and Antigens

An anti-MIC-1 mAb (MAB957, R&D Systems) was the capture antibody and was conjugated to polymer-coated p-Chips as previously described [21]. Recombinant MIC-1 protein (957-GD, R&D Systems) was used as an antigen and spiked in a 1:4 diluted pooled normal human male serum (Bioreclamation) in experiments designed for determining the standard curve. The recombinant protein was derived from Chinese Hamster Ovary cell line with an N-terminal 6-His tag. A biotinylated anti-MIC-1 Ab (BAF940, R&D Systems) was the detection antibody. The staining reagent was a streptavidin-phycoerythrin (SAPE) conjugate (Invitrogen).

### Serum Samples

A total of 70 de-identified serum samples were obtained from the Brady Urological Institute of the JHUSOM biorepository and consisted of 5 groups with 14 sera per group (normal, biopsy negative (Bx-ve), PCa patients with PSA < 2.5 ng/ml, PSA 2.5–10 ng/ml and PSA > 10 ng/ml). For normal group, the digital rectal examination (DRE), PSA test and the diagnoses were negative. Men in Bx-ve group had an abnormal total serum PSA of ≥ 4.0 ng/ml and/or a positive DRE exam and a recommended 12–14 core biopsy was performed at the Johns Hopkins Hospital (JHH) Urology clinic. The pathology interpreted diagnosis for Bx-ve group was negative. PCa patients underwent the same exams and the pathology diagnosis was positive. Gleason scores were available for 42 the PCa patients: 19 were GS 6, 14 GS 7, 5 GS 8 and 4 GS 9. The associated PSA levels and GS for each sample were provided by the JHUSOM biorepository.

### Prostate Cell Line Lysates

The PCa cell lines were grown and prepared for testing at the JHUSOM: PC3, DU145 & LNCaP and benign prostatic hyperplasia (BPH) cell line were grown in RPMI media supplemented with 10% fetal bovine serum (FBS) and normal prostate epithelial cell line PrEC in prostate epithelial cell growth medium (PrEGM; Lonza, Walkersville, MD, USA) in 12-well culture plates till 80–90% confluence. All cell lines used in this study originated from the ATCC in Rockville, MD, USA. After washing the cell monolayer with Hanks Balanced Salt Solution (HBSS), the proteins were extracted using Mammalian Protein Extraction Reagent (M-PER) (Thermo Fisher Scientific) and total protein levels were determined in the supernatant using BCA method (Thermo Fisher Scientific). The lysates were stored in aliquots at -80^°^C.

### p-Chips and Preparation of Immunoassay Kits

p-Chips were manufactured by PharmaSeq, Inc. (Monmouth Junction, NJ, USA). The properties of p-Chips and readers (either flow-based or hand-held) have been previously described [19–23]. Each MIC-1 assay kit consisted of 5 p-Chips conjugated with MIC-1 capture antibody placed in 500 μl test tubes. The IDs of the five chips were recorded in the assay file for each kit. The described kit was used for one sample.

### MIC-1 Immunoassay on p-Chips

#### Polymer Coating on p-Chips

The polymer coating on p-Chips was prepared by reacting aminopropyltriethoxysilane (APTS) and 3-glycidoxypropyl-trimethoxysilane (GPTS) as previously reported [21, 23]. The procedure placed both amino and hydroxyl groups within the polymer and on the surface of p-Chips. The amino groups were subsequently converted to carboxyl groups [21, 23]. All chemicals used for coating and carboxyl conversion were purchased from Sigma-Aldrich.

#### Biochemical Steps

A MIC-1 ELISA was performed on the polymer-coated p-Chips. The capture anti-human MIC-1 mAb was conjugated to the chip as previously described [21, 23]. In order to minimize serum interference, the serum samples were diluted to 1:4 in LowCross buffer (Candor Bioscience) and incubated with the anti-MIC-1 mAb-conjugated p-Chips for 1 hour at RT on a rotator. Five p-Chips were used for each serum sample. p-Chips were also incubated with a series of dilutions of recombinant MIC-1 protein prepared in 1:4 diluted pooled male serum to build the standard curve. After incubation, the p-Chips were washed with 200 μL of Tris-buffered saline with 0.05% Tween-20 (TBST) three times and subsequently incubated with 20 μL 5.0 μg/mL biotinylated anti-human MIC-1 polyclonal antibody for 1 hour at RT. The p-Chips were then washed with TBST three times, pooled and incubated with 10 μg/mL SAPE conjugate in TBST for 30 min at RT in the dark. After incubation, the p-Chips were washed with TBST three times and analyzed on the PharmaSeq flow reader.

#### Readout on Flow Reader

Typically, each MIC-1 concentration determination required 5 p-Chips. Since each p-Chip has a unique ID, multiple assays (approximately 20) were combined in a single run without loss of p-Chip identity. Analysis typically took 5–8 min with each p-Chip being read ≥20 times during multiple passes through the flow reader to reduce the coefficient of variation (CV).

To obtain the MIC-1 concentration in cell lysates, the recombinant MIC-1 protein was spiked into LowCross buffer (Candor Bioscience) at a series of concentrations to build the standard curve. Cell lysates were diluted to 300 μg/ml (total protein) using PBS and tested as described above in the serum-based assay.

### Statistical Analysis

MIC-1 concentrations in each group were compared using two-tailed unpaired t-test after passing the D’Agostino & Pearson omnibus normality test. The statistical significance level was set at P < 0.05. The two-tailed unpaired t-test and the D’Agostino & Pearson omnibus normality test were done in Prism (version 6.0). Receiver operator characteristic (ROC) curves were generated using the DeLong mathematical model [24] and compared in a two-tailed test. In order to generate the MIC-1/PSA ROC curve, log_10_ of the concentrations of MIC-1 and PSA were subjected to multivariate logistic regression analysis. The ROC curve analysis and mean/median calculation were done in MedCalc (version 12.7.8.0).

The limit of detection (LOD) is defined as the lowest detectable analyte concentration in the assay. To obtain the LOD, three standard deviations (SD) were added to the average normalized fluorescence intensity (NFI) of five replicates of the 0-standard.

## Results

### MIC-1 Is Highly Expressed in PCa Cell Line LNCaP

To validate the p-Chip-based MIC-1 assay, we first assessed the concentration of MIC-1 in selected PCa cell lines (LNCaP, PC3, DU145) and control cell lines, PrEC and BPH. We found that, as previously reported [17, 25], MIC-1 is highly expressed in androgen-sensitive LNCaP cells, whereas in the androgen-insensitive PC-3 and DU-145 cells the expression level was very low (S1 Fig). In addition, MIC-1 expression is low in the control BPH cells (S1 Fig) in agreement with the Kakehi findings [25]. In the control PrEC cells, MIC-1 expression was also ~8-fold less than that in the LNCaP cells.

### Serum Concentrations of MIC-1 in PCa Patients

A total of 70 serum samples from normal and PCa patients were tested. The LOD of the p-Chip-based MIC-1 assay was determined to be 0.09 ng/ml. The LOD value was initially calculated using fluorescence units, then extrapolated from the standard curve. The standard curve and detailed assay characteristics are summarized in S2 Fig.

**Fig 2:**
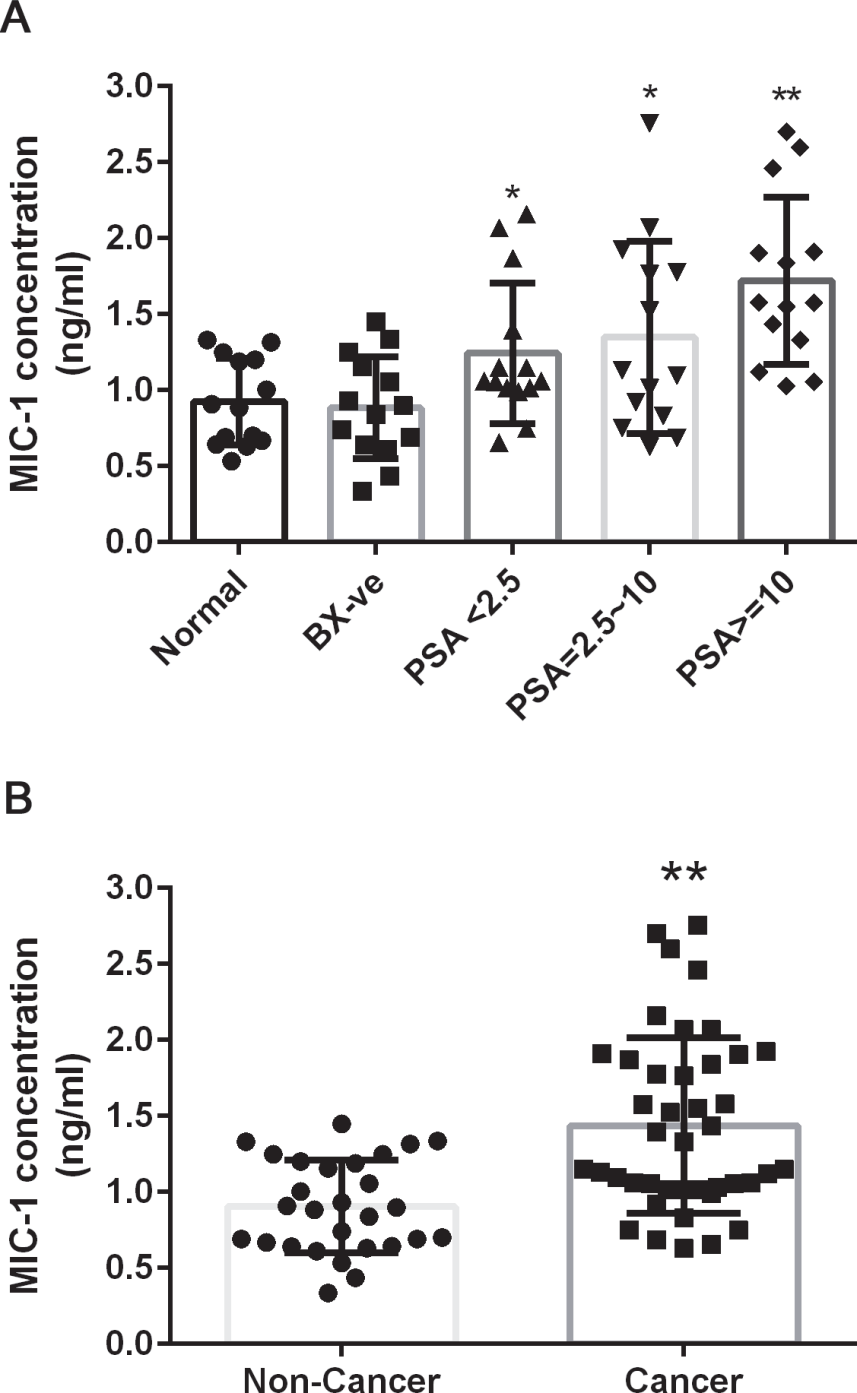
MIC-1 level in 70 serum samples. Individual MIC-1 concentration and average in each sample group (panel A) and combined nonPCa and PCa group (panel B) is shown. Error bar represents standard deviation. Asterisk indicates P<0.05 in two-tailed unpaired t-test when the PCa group is compared to nonPCa (normal or Bx-ve group). Double asterisks indicates P<0.01 in the two-tailed unpaired t-test. Bx-ve: biopsy negative.

In samples from the control group (normal) and Bx-ve patients the mean concentrations of MIC-1 were 0.93 ng/ml and 0.88 ng/ml, respectively (Table 1 and Fig 2), with no significant differences between the two groups (P = 0.726). The mean MIC-1 concentration in PCa patients with PSA <2.5 ng/ml was 1.24 ng/ml and significantly higher than in the normal (P = 0.039) and Bx-ve groups (P = 0.027). The mean MIC-1 concentrations in the other PCa patients groups (PSA 2.5–10 ng/ml and PSA > 10 ng/ml) were 1.35 ng/ml and 1.72 ng/ml, respectively, and also significantly higher than normal (P = 0.031 and P < 0.001, respectively) and the Bx-ve group (P = 0.022 and P < 0.001 respectively). When all the PCa cases and non-PCa cases were combined into two groups, the average MIC-1 concentration was significantly higher in the PCa patients than the group without PCa (0.90 ng/ml v.s. 1.44 ng/ml, P < 0.001). The MIC-1 serum concentration in each of the 70 samples can be found in S1 Table.

**Table 1:** Serum concentrations of MIC-1 and PSA in PCa patients.

		PSA				MIC-1			
	Number	Mean	Median	Range	95th percentile	Mean	Median	Range	95th percentile
Normal	14	1.03	0.80	0.25–2.30	2.30	0.93	0.90	0.53–1.33	1.33
Bx-ve	14	6.42	4.80	3.80–19.40	17.62	0.88	0.87	0.34–1.45	1.43
PSA <2.5	14	1.80	1.90	0.10–2.46	2.44	1.24	1.06	0.66–2.16	2.14
PSA 2.5–10	14	5.11	4.65	2.60–9.10	9.00	1.35	1.11	0.63–2.76	2.62
PSA ≥ 10	14	29.86	12.90	10.00–241.30	196.96	1.72	1.58	1.03–2.70	2.68
All nonPCa	28	3.72	3.05	0.25–19.40	11.39	0.90	0.89	0.34–1.45	1.35
All PCa patients	42	12.26	4.65	0.10–241.30	18.58	1.44	1.24	0.63–2.76	2.64

Concentration units: ng/ml. Bx-ve: biopsy negative.

### Combination of MIC-1 and PSA Improves PCa Diagnosis

**Fig 3:**
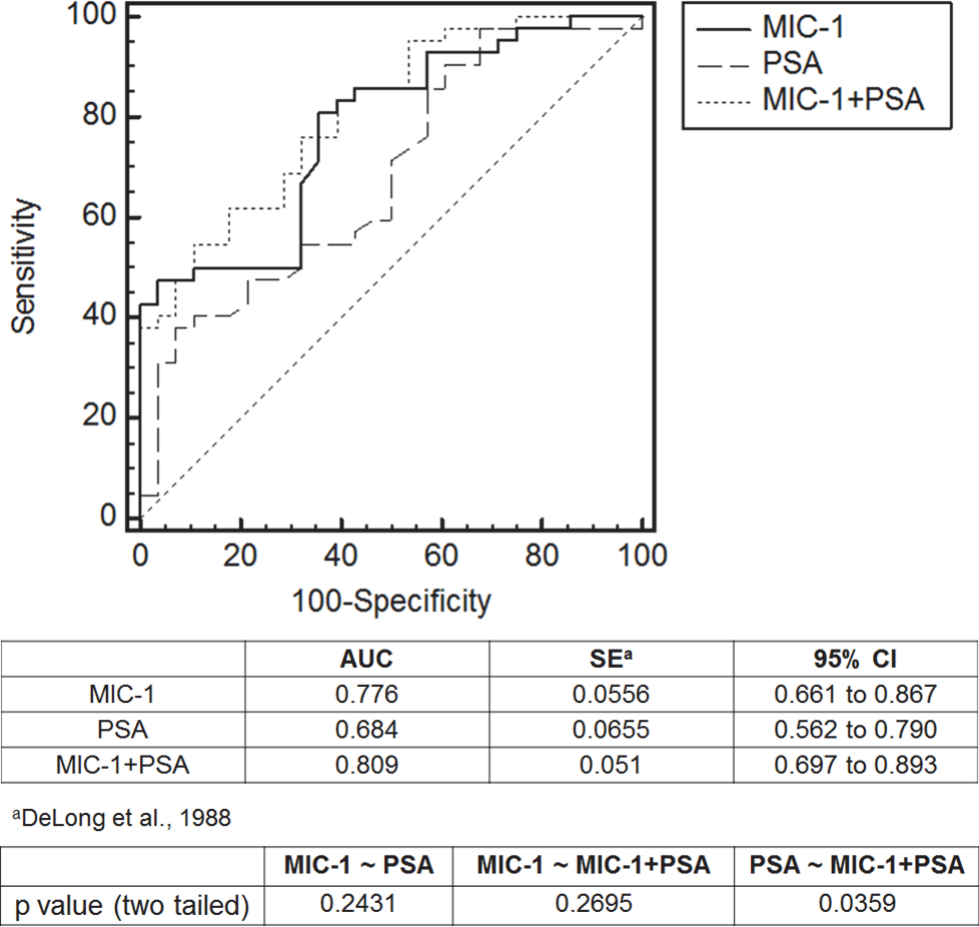
ROC curves for MIC-1, PSA or MIC-1+PSA.

The elevated MIC-1 level in PCa patient serum was correlated with PSA levels of the 70 serum samples (S1 Table) We therefore generated a PSA ROC curve and compared it to ROC curve for MIC-1 (Fig 3). The area under ROC (AUC-ROC) of MIC-1 was 0.776 and AUC-ROC for PSA is 0.684. However, the difference was not significant (P = 0.2431). Using multivariate logistic regression analysis for MIC-1 and PSA, we determined that a combination of the MIC-1 and PSA levels performed better in discriminating between non-PCa (normal and Bx-ve patients) and PCa. The AUC-ROC of MIC-1/PSA was 0.809 and significantly better than in the PSA only alone results (P = 0.0359) (Fig 3). By using the criterion of 0.494 for the logistic regression score of MIC-1-PSA as cutoff, we achieved the assay sensitivity of 83.3% and specificity of 60.7%. In comparison, the assay sensitivity was 54.8% and specificity was 57.1% if PSA = 4 ng/ml was used as a cutoff in the traditional PSA test, or assay sensitivity of 71.4% and specificity of 50% if PSA = 2.5 ng/ml was used as a cutoff. Thus our approach using serum MIC-1 as an additional biomarker to supplement the serum PSA results improves both sensitivity and specificity over serum PSA alone.

**Fig 4:**
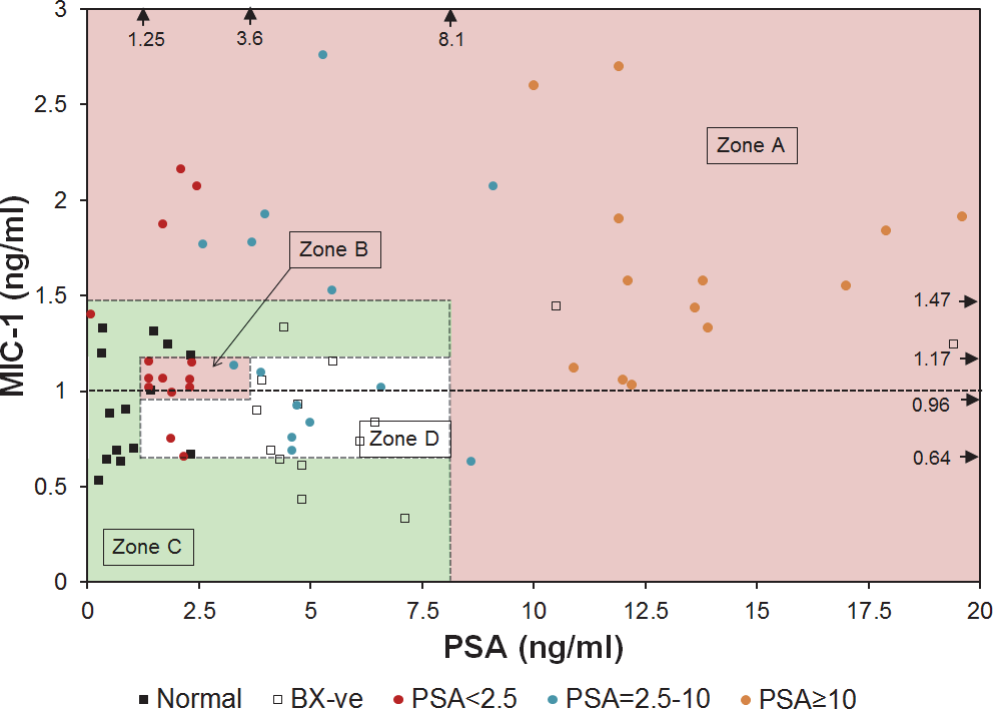
Red and green zones for PCa diagnosis in 2D plot of MIC-1 and PSA. For the ease of presentation, one patient with high PSA concentration of 241.3 ng/ml was not shown. Zone A: 24 PCa and 2 nonPCa, 92.3% cases are PCa; Zone B: 9 PCa and 1 nonPCa, 90% cases are PCa; Zone C: 1 PCa and 17 nonPCa, 94.4% cases are nonPCa; Zone D: 8 PCa and 8 nonPCa. Arrows near the top and right edges of the plot indicate the concentrations of biomarkers used to define the zones.

Another approach to utilize the results of both the MIC-1 and PSA assays was to generate a two dimensional (2D) plot in which the MIC-1 and PSA concentrations as the X- and Y-axes and then determining distinct zones based on the varying positive predictive value (PPV) for the sample set. Compared to traditional multi-factor linear formula, a 2D plot can offer better resolution to present data and can display more information. One such plot, based on all 70 samples plotted and involving four zones, is presented in Fig 4. Zones A, C and D correspond to high, low and unbiased PPV, respectively. The PPV is 92% (24/26) in Zone A, 5.6% (1/18) in Zone C and 50% (8/16) in Zone D. Surprisingly; a small isolated “hot” Zone B can be observed where the PPV to detect PCa is very high, 90% (9/10).

Combining Zones A and B into one category (high PPV zone) results in three risk categories for the samples as being in either high, unbiased or low PPV zones. The number of cases in the high PPV zone (Zones A and B) include 33 PCa (out of 42 total) and 3 non-PCa (out of 28 total), representing an assay sensitivity of 78.6% and specificity of 89.3%. Based on such these zone definitions, key characteristics of cancer determination are given in Table 2.

**Table 2:** Characteristics of cancer determinations.

Zone	Positive predictive value (PPV)	Purpose
A	92.3% (24/26)	PCa diagnosis
B	90% (9/10)	PCa diagnosis
C	5.6% (1/18)	PCa diagnosis
D	50% (8/16)	PCa diagnosis
A+B	91.7% (33/36)	PCa diagnosis
E	89.5% (17/19)	PCa prognosis, Gleason 6
F	78.6% (11/14)	PCa prognosis, Gleason 7

**Fig 5:**
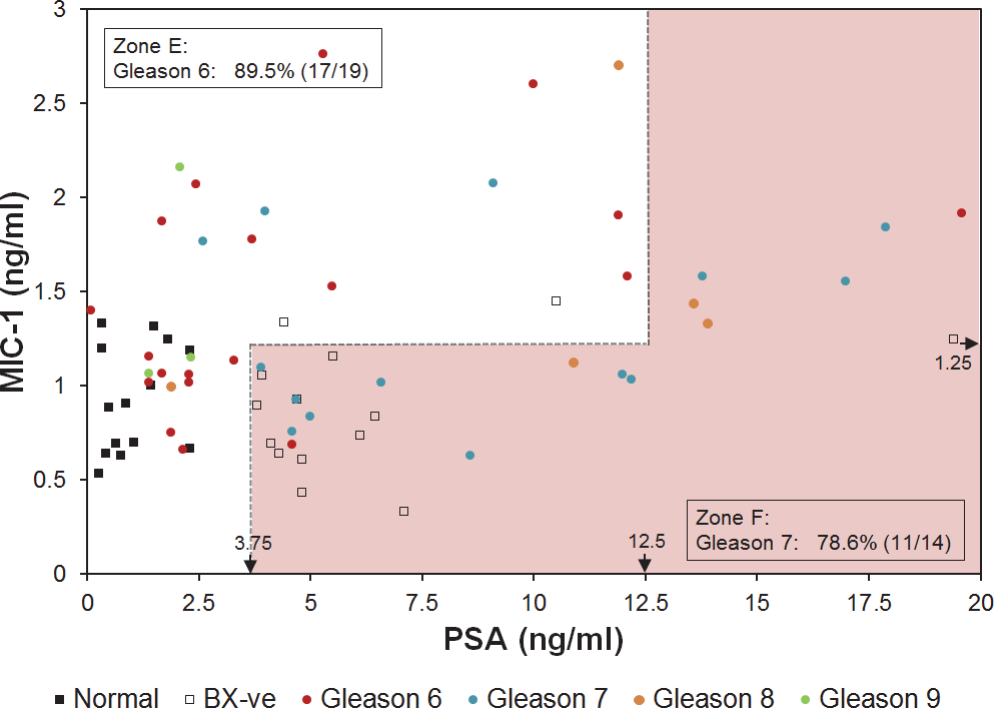
Red and green zones for PCa prognosis in 2D plot of MIC-1 and PSA. For the ease of presentation, one patient with high PSA concentration of 241.3 ng/ml was not shown. Zone E includes 89.5% (17/19) PCa patients with Gleason score 6. Zone F includes 78.6% (11/14) PCa patients with Gleason score 7. Arrows near the bottom and right edges of the plot indicate the concentrations of biomarkers used to define the zones.

The zones are defined in Fig 4 (A-D) and Fig 5 (E, F).

### Combination of MIC-1 and PSA Discriminates Gleason Scores 6 and 7

Gleason scores (GS) were analyzed for the distribution of GS on a MIC-1/PSA plot. In the 70 samples investigated, two scores dominated: GS 6 (19 samples) and GS 7 (14 samples). We found that a correlation exists between the MIC-1/PSA concentrations and the two categories of GS. Two zones, corresponding to GS 6 and 7, respectively, are shown in Fig 5, and the frequencies of GS 6 and 7 presented in S1 Table. A high-frequency zone for GS6 is Zone E in Fig 5 in which 89.5% (17/19) of the cases are the PCa patients with GS 6. In contrast, a high-frequency zone for GS 7 is Zone F (also in Fig 5). It includes 78.6% (11/14) of PCa patients with GS 7. The results are summarized in Table 2. Points corresponding to GS 8 and 9 are distributed in the upper half of the graph, with a preference for the hot spot (Zone B in Fig 4).

## Discussion

### Value of Improved PCa Assay

Today, 1 in 6 men will be diagnosed with PCa in their lifetime, but only 1 in 37 men will die from it [1, 26]. The landmark European Randomized Study of Screening for PCa (ERSPC) study [27] suggested that to save 1 life over an 11 year period, 1055 men will need to be screened for PSA and 37 men treated. One of the central questions in PCa remains: “Are we identifying men with small PCa tumors who may never present with “significant disease” in their lifetime and, therefore, may not require treatment and can be monitored annually [28–30]?” A second major problem relates to the over-treatment of PCa due to screening. In May 2012, the US Preventive Task Force issued a recommendation against PSA based screening for PCa, concluding that it causes more harm than good [28]. While difficult to approximate, this over-diagnosis of PCa has been estimated to occur for 23–56% of men, and this number will increase as our population ages [4, 31, 32]. Thus, the significance of an assay and the analysis method (Fig 4) that can improve the specificity of the PCa detection, such as an assay described in this paper, is high. Also, this study has high impact for associating Gleason scores 6 and 7 with MIC-1 and PSA concentrations (Fig 5), of significance in determining the treatment.

### MIC-1 as a Highly Significant Biomarker for Serum-based PCa Test

The main conclusion of this study is that adopting the MIC-1/PSA immunoassay as a PCa screen significantly improves the specificity of the PCa determination compared with the PSA alone test. Using a set of 70 serum samples we found that the PPV was 91.7% in the combined MIC-1/PSA assay, compared with 65.7% if 4 ng/ml was used as cutoff or 66.7% if 2.5 ng/ml was used as cutoff for the PSA only assay (Table 2 and Fig 4). The improvement is seen whether or not the “hot” Zone B is used, although it is more pronounced when Zone B is included into the analysis. This paper expands on previous findings that demonstrated that the use of serum MIC-1 increases diagnostic specificity for PCa determination in cases where a GS was ≥7 [33], and that serum MIC-1 concentration is correlated with PCa prognosis [34]. We have demonstrated an even greater improvement of assay specificity as a result of knowing the concentration of MIC-1 in addition to PSA, and (2) indicating a possible “PCa hot spot” (high risk) on the MIC-1/PSA plot (Fig 4). Moreover, for any given patient, we correlate the concentrations of MIC-1 and PSA with Gleason scores (Fig 5). It is worth noting that the sample size of this study is relatively small. The cut-off values to assign different zones (Figs 4 and 5) are determined empirically but not statistically due to the limitation of this study. Here we demonstrated the concept of using zones in a 2D plot for a better PCa detection. A more accurately defined zone map can be determined in future studies with larger sample size.

We find it puzzling, however, that in contrast to our results, previous studies from other groups reported that the PCa group had lower serum MIC-1 levels than the normal group [33, 35]. In another published MIC-1 prognostic study [34] though MIC-1 serum levels predicted poor cancer-specific survival as the MIC-1 serum levels increased in different patient groups. The reasons for the discrepancy are still under investigation. It is likely due to different physiological and genetic background of patients selected in the two studies since MIC-1 has been found to have pleiotropic roles in inflammation, cancer and metabolism [36] and its expression can be modulated by age [33] and numerous natural and pharmacological compounds [37]. In addition, the genetic variation of MIC-1 gene can also be correlated to MIC-1 expression and serum levels [34]. Therefore other health and genetic background factors should be carefully considered in order to compare studies.

### Two Biochemical Signatures of Prostate Cancer

The current study raises the intriguing possibility of having two distinct biochemical signatures. The first signature comprises high-PSA, high-MIC-1 values while the second—biomarker concentrations in the hot spot (low to medium PSA, medium MIC-1 values). This might possibly correspond to two distinct forms of prostate cancer within the adenocarcinoma category, leading to potentially different treatments and therapies.

Interestingly, the distribution of the PSA levels in the general population according to the published data [38] show a clear second peak ("bump") in the range of PSA values of 3.0–4.5, which might be indicative of two components contributing to the observed PSA concentration in the patient sera. Further studies are needed.

### Gleason Scores on the MIC-1/PSA Plot

A significant question can be asked as to whether the MIC-1/PSA plot (Fig 5) can lead to a prediction of the GS if a patient has prostate cancer. We showed that, for any given sample from a PCa patient whose biopsy GS is 6 or 7, we can confirm with 78.6% accuracy whether it is GS 6 or GS 7. This may also be useful post-operatively to predict that the PCa may actually be a GS 7 instead of a GS 6. Clearly, a placement of the MIC-1/PSA serum concentration point in the Zone F on the plot may be indicative of the presence of high risk PCa since it correlates with higher GS. Points corresponding to Gleason scores 8 and 9 are distributed in the upper half of the plot, with a preference for the hot spot.

### Performance of p-Chip Platform in Immunoassay and the Future

The MIC-1 assay described above was implemented on the automated p-Chip PharmaSeq immunoassay platform which has major advantages. First, the requirement for the sample volume is far less than in a routine 96-well based ELISA (e.g., a total of 40 μL). Second, several assay kits (≥20) can be combined and analyzed at the same time. Third, the platform is designed for multiplex assays, thus adding an assay (e.g., in this case, PSA) could be readily done. Other benefits (silicon as solid phase, ability to modify the surface to enhance fluorescence) have been previously described [20, 21, 23]. The results provide a clear direction for further clinical testing and analyses, including conducting an investigation based on a much larger set of samples/patient cohorts and controls with a clearly defined training and test set. It would be worthwhile to study trajectories on the MIC-1/PSA plot as the PCa develops over time for selected patients, or MIC-1 dynamics, such as velocity tracking changes in the MIC-1 and PSA. The approach has high potential for improving PCa diagnosis and prognosis.

## Supporting Information

S1 Fig Expression of MIC-1 in different cell line. Cell lysates from the cultured cells were diluted to 300 μg/ml (total protein) and tested by the p-Chip-based assay. Five replicates were included in each sample. Error bars indicate standard deviation.
(TIF)

S2 Fig Assay characteristics of p-Chip based MIC-1 assay. (TIF)

S1 Table Levels of MIC-1 and PSA in 70 serum samples. Concentration unit: ng/ml. Bx-ve: biopsy negative.
(DOC)
