# Supplementary figures and images for: Macrophage Inhibitory Cytokine 1 Biomarker Serum Immunoassay in Combination with PSA Is a More Specific Diagnostic Tool for Detection of Prostate Cancer
Source: PLoS One. 2015 Apr 8;10(4):e0122249. doi: 10.1371/journal.pone.0122249 (PMC4390224; doi:10.1371/journal.pone.0122249)

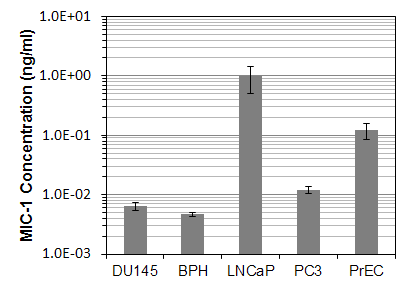

Supplement: S1 Fig — Cell lysates from the cultured cells were diluted to 300 μg/ml (total protein) and tested by the p-Chip-based assay. Five replicates were included in each sample. Error bars indicate standard deviation. (TIF) [file pone.0122249.s001.tif]

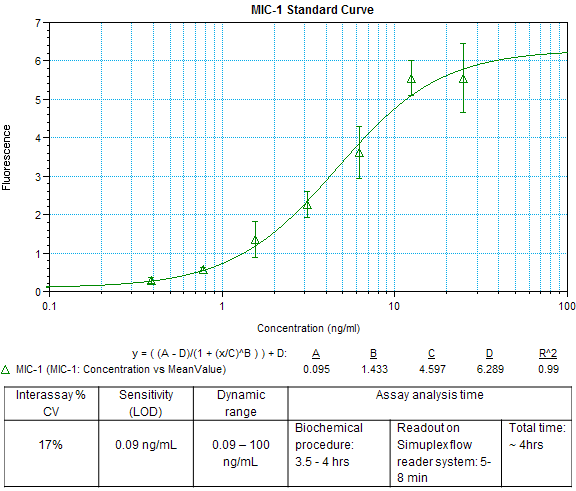

Supplement: S2 Fig — (TIF) [file pone.0122249.s002.tif]
