# Supplementary material for: Macrophage Inhibitory Cytokine 1 Biomarker Serum Immunoassay in Combination with PSA Is a More Specific Diagnostic Tool for Detection of Prostate Cancer
Source: PLoS One. 2015 Apr 8;10(4):e0122249. doi: 10.1371/journal.pone.0122249 (PMC4390224; doi:10.1371/journal.pone.0122249)
Supplement: S1 Table — Concentration unit: ng/ml. Bx-ve: biopsy negative. (DOC) [file pone.0122249.s003.doc]

|  |  | **Sample ID** | **PSA** | **Gleason** | **MIC-1** |
| --- | --- | --- | --- | --- | --- |
| **Normal** | 1 | 8430 | 2.3 |  | 0.668 |
|  | 2 | 7033 | 1.8 |  | 1.248 |
|  | 3 | 6990 | 2.3 |  | 1.188 |
|  | 4 | 5812 | 0.74 |  | 0.632 |
|  | 5 | 5810 | 0.86 |  | 0.908 |
|  | 6 | 5807 | 0.42 |  | 0.644 |
|  | 7 | 5805 | 0.49 |  | 0.884 |
|  | 8 | 5804 | 0.32 |  | 1.200 |
|  | 9 | 6844 | 1.41 |  | 1.004 |
|  | 10 | 6847 | 1.03 |  | 0.702 |
|  | 11 | 6848 | 0.25 |  | 0.534 |
|  | 12 | 6851 | 0.33 |  | 1.332 |
|  | 13 | 6886 | 1.48 |  | 1.315 |
|  | 14 | 6892 | 0.64 |  | 0.692 |
|  | Mean |  | 1.026 |  | 0.925 |
|  | STDEV |  | 0.718 |  | 0.286 |
|  | Mean (PSA<1) |  | 0.506 |  | 0.853 |
|  | STDEV (PSA<1) |  | 0.219 |  | 0.286 |
| **BX-ve** | 1 | 9270 | 6.1 |  | 0.740 |
|  | 2 | 9226 | 5.5 |  | 1.156 |
|  | 3 | 9217 | 3.9 |  | 1.056 |
|  | 4 | 9216 | 6.44 |  | 0.840 |
|  | 5 | 8985 | 4.4 |  | 1.336 |
|  | 6 | 8971 | 10.5 |  | 1.448 |
|  | 7 | 8928 | 19.4 |  | 1.248 |
|  | 8 | 8885 | 4.8 |  | 0.436 |
|  | 9 | 11122 | 4.1 |  | 0.692 |
|  | 10 | 11129 | 7.1 |  | 0.335 |
|  | 11 | 11240 | 4.8 |  | 0.611 |
|  | 12 | 11241 | 4.3 |  | 0.642 |
|  | 13 | 11488 | 4.7 |  | 0.930 |
|  | 14 | 11489 | 3.8 |  | 0.898 |
|  | Mean |  | 6.417 |  | 0.883 |
|  | STDEV |  | 4.132 |  | 0.335 |
| **PSA<2.5** | 1 | 8721 | 1.39 | 6 | 1.152 |
|  | 2 | 8665 | 1.89 | 6 | 0.748 |
|  | 3 | 8623 | 2.46 | 6 | 2.068 |
|  | 4 | 7687 | 1.4 | 6 | 1.016 |
|  | 5 | 7101 | 0.1 | 6 | 1.396 |
|  | 6 | 7038 | 2.3 | 6 | 1.056 |
|  | 7 | 6610 | 1.9 | 8 | 0.992 |
|  | 8 | 6202 | 1.7 | 6 | 1.868 |
|  | 9 | 11120 | 1.4 | 9 | 1.061 |
|  | 10 | 11124 | 2.1 | 9 | 2.159 |
|  | 11 | 11131 | 1.7 | 6 | 1.061 |
|  | 12 | 11466 | 2.17 | 6 | 0.656 |
|  | 13 | 11505 | 2.3 | 6 | 1.016 |
|  | 14 | 11511 | 2.35 | 9 | 1.148 |
|  | Mean |  | 1.797 |  | 1.243 |
|  | STDEV |  | 0.613 |  | 0.465 |
| **PSA=2.5-10** | 1 | 9295 | 5.5 | 6 | 1.524 |
|  | 2 | 9288 | 3.7 | 6 | 1.776 |
|  | 3 | 9287 | 4.6 | 7 | 0.752 |
|  | 4 | 9286 | 5 | 7 | 0.832 |
|  | 5 | 9285 | 2.6 | 7 | 1.764 |
|  | 6 | 9246 | 4 | 7 | 1.924 |
|  | 7 | 9195 | 5.3 | 6 | 2.756 |
|  | 8 | 9194 | 3.3 | 6 | 1.132 |
|  | 9 | 11467 | 9.1 | 7 | 2.070 |
|  | 10 | 11468 | 4.6 | 6 | 0.685 |
|  | 11 | 11469 | 8.6 | 7 | 0.628 |
|  | 12 | 11472 | 3.9 | 7 | 1.095 |
|  | 13 | 11473 | 4.7 | 7 | 0.922 |
|  | 14 | 11487 | 6.6 | 7 | 1.013 |
|  | Mean |  | 5.107 |  | 1.348 |
|  | STDEV |  | 1.870 |  | 0.633 |
| **PSA≥10** | 1 | 8666 | 12 | 7 | 1.056 |
|  | 2 | 8646 | 17 | 7 | 1.552 |
|  | 3 | 8641 | 241.3 | 9 | 2.460 |
|  | 4 | 6008 | 12.1 | 6 | 1.576 |
|  | 5 | 6003 | 10 | 6 | 2.600 |
|  | 6 | 5941 | 13.6 | 8 | 1.436 |
|  | 7 | 5744 | 11.9 | 6 | 1.904 |
|  | 8 | 5696 | 11.9 | 8 | 2.700 |
|  | 9 | 11355 | 13.8 | 7 | 1.579 |
|  | 10 | 11403 | 10.9 | 8 | 1.121 |
|  | 11 | 11426 | 17.9 | 7 | 1.838 |
|  | 12 | 11443 | 19.6 | 6 | 1.912 |
|  | 13 | 11540 | 13.9 | 8 | 1.330 |
|  | 14 | 11619 | 12.2 | 7 | 1.030 |
|  | Mean |  | 29.864 |  | 1.721 |
|  | STDEV |  | 60.918 |  | 0.551 |
| **PCa patients all** | Mean |  | 12.256 |  | 1.437 |
|  | STDEV |  | 36.586 |  | 0.579 |
| **Gleason 6** | Mean |  | 4.916316 | 6 | 1.468551 |
| **Gleason 7** | Mean |  | 8.714286 | 7 | 1.289614 |
| **Gleason 8** | Mean |  | 10.44 | 8 | 1.515763 |
| **Gleason 9** | Mean |  | 61.788 | 9 | 1.706847 |
